# Assessment of the antifungal efficacy of whey fermentate alone or in combination with citrus extract to control *Aspergillus flavus* mold in semi-moist pet food for dogs

**DOI:** 10.3389/fmicb.2023.1188834

**Published:** 2023-11-01

**Authors:** Samuel Kiprotich, Janak Dhakal, Cynthia Rasmussen, Greg Aldrich

**Affiliations:** ^1^Department of Grain Science and Industry, Kansas State University, Manhattan, KS, United States; ^2^Department of Agriculture, Food and Resource Sciences, University of Maryland Eastern Shore, Princess Anne, MD, United States; ^3^Kerry, Americas Region, Food Protection and Fermentation, Beloit, WI, United States

**Keywords:** antifungals, semi-moist, water activity, *Aspergillus flavus*, fermented whey, citrus extracts, pet food

## Abstract

Semi-moist pet foods contain moisture levels ranging from 15 to 40%, making them ideal for mold growth and mycotoxin production. To control spoilage, synthetic mold inhibitors such as potassium sorbate have been used, but consumers prefer “natural” preservatives. Whey fermentate (WPF) is an efficient antifungal, but it requires large doses. Therefore, the objective of this study was to determine the antifungal effect of WPF alone or in combination with citrus extract oil (CEX) against *Aspergillus flavus* in semi-moist pet food. Nutritionally complete semi-moist pet foods were produced with WPF alone [0.25, 0.5, and 1.0% (w/w)] and in combination with CEX; 0.25% WPF+ 0.015% CEX, 0.25% WFP+ 0.15% CEX, 0.5% WPF+ 0.015% CEX, and 0.5% WFP+ 0.15% CEX (w/w). The negative control (NC) contained no antifungal additive and the positive control (PC) had potassium sorbate (0.1% w/w). The semi-moist pet food was thermally formed and was cut into 3 cm × 3 cm square pieces. Individual food pieces were inoculated with 0.1 mL of *Aspergillus flavus* (ATCC 204304) to achieve a final concentration of ~5.0 log CFU/piece. The inoculated pieces were individually incubated at 25°C. Fungal analysis was performed on day 3, 6, 9, 12, 15, 18, 21, 24, and 28 by surface plating on Potato Dextrose Agar (PDA) followed by incubation at 25°C for 72 h. The total log reductions were calculated by subtracting the initial inoculum from the final log counts on day 28. Higher log reductions of *Aspergillus flavus* (*p* < 0.05) were observed when WPF at 0.25 and 0.5% was combined with 0.15% CEX compared to when 0.015% CEX was used individually. All treatments were different from the NC (*p* < 0.05). Citrus extract at 0.15% potentiated the antifungal effect of WPF at 0.5% to give a similar log reduction (*p* > 0.05) to WPF at 1.0% in the food. In conclusion, CEX potentiated the antifungal efficacy and reduced the dose of WPF required to control *Aspergillus flavus* in semi-moist dog food.

## Introduction

Semi-moist pet foods belong to the category of minimally processed diets and are characterized by their high moisture content of 15 to 40% and water activity of 0.60 to 0.85 (Rolinec et al., 2016; Kiprotich et al., 2021; Deliephan, 2022). Semi-moist pet foods have increased in popularity globally due to their quality and sensory attributes, which are similar to those of fresh diets. They are edible without hydration and have a prolonged shelf life (Qiu et al., 2019). Semi-moist pet foods are a diverse category of diets that are mostly classified according to the macro ingredients used to manufacture the base rations, such as cereals, meats, or dairy products. These foods are often given to companion animals as treats for training and reinforcement of positive behavior.

Commercially, semi-moist pet foods such as chewy dog treats are produced through partial drying or osmotic drying using humectants. Diets are also formulated with humectants to enhance shelf life (Deliephan, 2022). The desired water activity in semi-moist or intermediate-moisture pet food is achieved through the application of salt, sugar, glycerol, or propylene glycol, which immobilize and bind free water, which is necessary for the formation of these diets (Fellows, 2009). Upon formulation, the ingredients are assembled, and the food is manufactured through baking, cooking, extrusion, or thermal dehydration (Fellows, 2009).

Semi-moist pet foods are attractive to many pet owners because they are ready to eat (RTE), highly nutritious, palatable, and microbially safe (Severini et al., 2008). However, semi-moist pet foods are susceptible to mold growth and proliferation due to their high moisture content and water activity, which are favorable to the survival of spoilage and toxigenic fungi (Deliephan et al., 2023). The proliferation of fungi in semi-moist pet foods causes spoilage, which leads to economic losses for pet food manufacturers who must carefully dispose of contaminated products. Moreover, some fungal species in genera like Aspergillus, Penicillium, Fusarium, and Alternaria biosynthesize toxic secondary metabolites, mycotoxins (Deliephan, 2022). Examples of mycotoxins include, but are not limited to, ochratoxins, deoxynivalenol, zearalenone, with aflatoxins being the most important toxins in the semi-moist pet food segment (Pitt and Hocking, 2009). Ingestion of mycotoxins by humans and animals causes mycotoxicosis, which might present as acute or chronic cytotoxicity, hepatotoxicity, mutagenicity, or carcinogenicity (Janik et al., 2020). Restuccia et al. (2019) reported that pets experiencing aflatoxin poisoning exhibited symptoms such as sluggishness, loss of appetite, vomiting, jaundice, or diarrhea. Therefore, it is crucial that the microbial safety of semi-moist pet food is adequate, and the ensuing products are safe for animal consumption.

Thus, to enhance the microbial safety of semi-moist pet foods, moisture and water activity must be strictly controlled using humectants like propylene glycol, glycerol, or sugar (Zicker, 2008). This is because the final moisture content in these foods’ ranges from 25 to 35%, which provides sufficient “active water” required for mold proliferation (Zicker, 2008; Kiprotich et al., 2021). To control mold proliferation, antimycotic agents such as potassium sorbate, calcium propionate, and organic acids (e.g., butyric acids, parabens, etc.) are formulated into semi-moist pet foods during the manufacturing process (Kiprotich et al., 2021). However, there is an increasing demand for pet foods considered “natural” and free of synthetic food additives or preservatives, which are less desirable to pet owners who prefer their animals consume clean-label foods (Kiprotich et al., 2021).

Whey is a liquid by-product of cheese manufacturing obtained after coagulation of casein proteins (Brandelli et al., 2015). It is a cheap carbon source for lactic acid bacteria (LAB), which require nutrients for fermentation. Whey is rich in lactose, soluble proteins, lipids, and minerals essential for the growth and multiplication of LAB (Panesar et al., 2007; Koutinas et al., 2009; Prazeres et al., 2012). The fermentation of whey using LAB leads to the production of important biomolecules such as bacteriocins. These are small heat-stable peptides of bacterial origin with antimicrobial and immune functions (Cotter et al., 2005). The fermentation process also produces several metabolites with antifungal properties, such as organic acids (acetic, lactic, formic, and propionic acids), benzoates, polyphenols, diacetyl, and short-chain fatty acids (Gerez et al., 2013; Cortés-Zavaleta et al., 2014; Miao et al., 2014; Gamba et al., 2015; Luz et al., 2020; Izzo et al., 2020a,b).

Given the increasing demand for pet foods formulated without synthetic preservatives, there is a need to explore natural and sustainable antifungal food additives to control mold in semi-moist products. Recent studies by Luz et al. (2020) show that whey fermented with *Lactobacillus plantarum* was able to extend the shelf life of pita bread when used as a substitute for calcium propionate. Preliminary studies by Kiprotich et al. (2021) reported an extension in shelf life and inactivation of *Aspergillus flavus* inoculated in semi-moist pet food treated with whey permeate fermentate (WPF) as a substitute for potassium sorbate. However, preliminary studies have demonstrated that higher doses of WPF were required to completely inactivate the *A. flavus*, but this caused significant negative changes in product quality and palatability. Thus, there was a need to potentiate the antifungal properties of WPF.

Citrus extracts (CEX) demonstrated limited antifungal activity when used alone to control *A. flavus* in semi-moist pet food, but preliminary studies showed some potentiation effect when combined with WPF. The hypothesis of this study was that WPF when used in combination with CEX would result in an additive or potentiation effect and thus would reduce the dose of WPF required to inhibit mold proliferation. To the authors’ knowledge, the antifungal properties of WPF and CEX have not been demonstrated alone, or in combination at smaller doses. Therefore, the objective of this study was to investigate the antifungal efficacy of WPF alone or in combination with CEX when challenged against *Aspergillus flavus* artificially inoculated in semi-moist pet food. The secondary objective was to determine the decimal reduction times (D-Values) and the effect of these antimycotic additives on the shelf life of semi-moist pet food for dogs.

## Materials and methods

### Preparation of *Aspergillus flavus* cultures

Cultures of *Aspergillus flavus* (ATCC 15548) were procured from the American Type Culture Collection (ATCC, Manassas, VA, United States) and frozen at −80°C. Working cultures were prepared by thawing and streaking the frozen inoculum on potato dextrose agar (PDA) plates using an inoculation loop. The streaked PDA plates were then incubated at 25°C for 72 h. *Aspergillus flavus* colony forming units were then scrapped using an inoculation loop and suspended in 10 mL of potato dextrose broth (PDB) and incubated at 25°C for 72 h. The final concentration of *A. flavus* culture was then determined by serial dilution and spread plating 0.1 mL aliquots on PDA, followed by incubation at 25°C for 72 h.

### Experimental design and treatments

The treatments in this study were determined from the preliminary experiments published in the extension report by Kiprotich et al. (2021). This experiment was a completely randomized design as the treatments were randomly assigned to the semi-moist pet food. The treatments consisted of a positive control (PC) that contained 0.1% (w/w) potassium sorbate as a standard industrial mold inhibitor, a negative control (NC) that did not contain any treatments and was meant for comparison with other treatments. The treatments included whey fermentate, Kerry Inc., Beloit, Wisconsin (WPF) alone or in combination with citrus extract, Kerry Inc., Beloit, Wisconsin (CEX) for *N* = 9. For the WPF, the maximum allowable concentration in the base ration formula was 1.0% (w/w) and the CEX was 0.15% as had been directed by the product manufacturer. The WPF and CEX were all obtained from Kerry, Americas Region, Beloit, Wisconsin for only research purposes. The treatments were replicated thrice. The semi-moist pet foods were formulated and thermally formed in an oven with the treatments described above.

### Preparation of semi-moist pet food

A nutritionally complete, semi-moist pet food was formulated to mimic a commercial product. All ingredients were weighed according to the formula (Table 1) to produce 2 kg batches of product. Dry ingredients including the respective treatments were mixed thoroughly followed by addition of wet ingredients and blended using a 3.8 kg planetary mixer (KitchenAid Portable Appliances, St. Joseph, MI) for 10 min at 50 RPM. A 1 cm thick layer of the batter was spread on a baking tray and thermally formed in a convention oven at 177°C for 10 min. The thermally formed product was cooled and then cut into uniform pieces (3 × 3 × 1 cm^3^) using a stainless-steel pizza knife. The food pieces were individually and aseptically transferred to sterile stomacher bags and refrigerated prior to inoculation.

**Table 1:** An in-house nutritionally complete formula* that was used for the preparation of semi-moist pet food.

Ingredient	%
Water	18.0
Corn	12.6
Chicken by-product meal	12.4
Corn gluten meal	12.4
Glycerin 99.7% USP	12.5
Wheat flour	5.8
Chicken fat	5.8
Corn syrup	5.0
Gelatin 250 bloom	2.5
Rice flour	4.3
Soybean meal	4.3
Molasses	1.0
Dry digest (flavoring)	0.5
Salt	0.5
Dicalcium P	1.4
Vitamin premix	0.2
Potassium Cl	0.2
L-Lys	0.1
Trace mineral premix	0.1
Calcium carbonate	0.1
Choline Cl (60%)	0.1
Potassium sorbate	0.1
Antioxidant	0.1

*Base formula for the positive control that was adopted and modified to produce 9 treatments of semi-moist pet food that contained different mold inhibitors and levels: WPF at 0.25, 0.5 and 1.0% (w/w). The WPF at 0.25 and 0.5% was combined with CEX at either 0.015% or 0.15% (w/w) (Kerry Ingredients, Beloit, WI). There was a positive control, which contained 0.1% potassium sorbate and a negative control without antimycotics.

### Fungal challenge study

The semi-moist pet food was inoculated an hour after thermal formation by delivering a 0.1 mL aliquot of *Aspergillus flavus* inoculum suspended in potato dextrose broth (PDB) to individual food pieces to achieve a final concentration of ~5.0 Log CFU/piece. The inoculum was spread evenly using a sterile L-shaped rod. The inoculated pieces were then stored in an incubator set at 25°C until analysis. The fungal concentration of the inoculum was determined by plating 0.1 mL of the inoculum on PDA plates and incubated at 25°C for 72 h. Fungal analysis was performed on day 0, 3, 6, 9, 12, 15, 18, 21, 24, and 28. For analysis, 100 mL of 0.1% peptone water was added to the food pieces in the stomacher bag and pummeled in the stomacher machine (Seward, Islandia, NY, USA) at medium speed for 1 min. Serial dilution, plating on PDA plates and incubation at 25°C for 72 h followed subsequently. The resulting colony forming units were enumerated and reported as log CFU/piece and then log reductions were calculated by subtracting the final log counts of the pathogens from the initial concentration of the inoculum.

### Determination of D-values of each treatment

The *D*-values (time of exposure to treatment that results in 90% reduction in viable counts of *Aspergillus flavus*) were determined by plotting the log number of surviving organisms per sample (inoculated semi-moist pet food pieces) against exposure time (days) using spreadsheets software (Microsoft Excel 2000 Software; Microsoft Inc., Redmond, WA). Using linear regression analysis, the line of best fit for each set of data was determined. The D-value was evaluated by calculating the negative reciprocal of the slope of the regression line for each treatment.

### Shelf life studies (days-to-mold study)

The shelf life study was conducted as a ‘days-to-mold’ experiment. Uninoculated semi-moist pet food pieces from each of the 9 treatments were stored in an incubator set at 25°C and a relative humidity (RH) of 45%. Six semi-moist pet food pieces from each individual treatment were placed into a sterile, self-sealing stomacher bag, including the negative control, and then stored in an incubator. The food pieces were monitored each day for any visible indicators of mold growth. The days-to-mold were considered as the total number of days it took for any food pieces within each treatment to develop visible signs of mold growth.

### Statistical analysis

One-way ANOVA was used to separate the log reduction and D-value means of the semi-moist pet foods that contained different antimycotic treatments. The means obtained from the log reduction and D-values of the food pieces treated with antimycotic agents were evaluated for significant differences at a 5% significance between the treatments using Tukey’s test (*p* ≤ 0.05), respectively. The variability in the data is expressed as the standard error of the means (SEM). Data were analyzed using JMP Pro version 16.1 statistical software (SAS Institute, Inc., Cary, NC).

## Results

### Fungal challenge study

The results from the fungal challenge study are reported on Table 2 in two formats; first in Log CFU reductions per piece of inoculated semi-moist pet food, which is the difference between initial mold count and the final mold count enumerated on day 28. The Log CFU reductions have then been reported as percent (%) log reductions for easier interpretation of the log reductions. There was a significant log reduction (*p* ≤ 0.05) for the PC treatment (0.1% Potassium sorbate) compared to the NC (contained no WPF or CEX treatments alone or in combination) as they had a reduction of 89.13 and 79.43%/piece, respectively (Table 2). There were significant log reductions in the WPF only treatments as their inclusion levels increased from 0.25 to 1.0% (w/w) (*p* < 0.05) in the base ration formula. Combinations of 0.25% or 0.5% WPF with 0.015% CEX did not result in any significant log reduction (*p* > 0.05) compared to when WPF was used alone. However, when CEX was increased 10-fold to 0.15% and added to 0.25 and 0.5% WPF treatments, there was an increase in log reduction (*p* < 0.05) of *Aspergillus flavus* compared to when WPF was used alone without CEX. Furthermore, combinations of 0.5% WPF and 0.15% CEX resulted in a log reduction of *A. flavus* not significantly different (*p* > 0.05) from the 1.0% WPF treatment alone. Overall, the log reductions in the fungal challenge study were higher (*p* < 0.05) than the NC except for two treatments (0.25% WPF alone and 0.25% combined with 0.015% CEX). The PC treatment which contained potassium sorbate as the standard industrial mold inhibitor, had log reductions comparable (*p* > 0.05) to 0.25% WPF alone or when it was combined with CEX at 0.015% or 0.15%. Increase of CEX from 0.015 to 0.15% resulted in increased log reductions (*p* < 0.05) when combined with WPF in the semi-moist pet food.

**Table 2:** Total log Reduction of *Aspergillus flavus* when exposed to semi-moist pet foods containing whey fermentate (WPF) alone or in combination with citrus extract (CEX) 28 days post-inoculation, incubated at 25°C.

Treatment level (%)	Log Reduction^1^	% Log Reduction
NC	0.7^E^	79.43
PC (0.1% potassium sorbate)	1.05^CD^	91.09
0.25% WPF	0.82^DE^	83.18
0.5% WPF	1.33^BC^	95.49
1.0% WPF	2.58^A^	99.74
(0.25% WPF + 0.015%% CEX)	0.89^DE^	87.38
(0.50% WPF + 0.015% CEX)	1.45^B^	96.77
(0.25% WPF + 0.15% CEX)	1.32^BC^	95.37
(0.50% WPF + 0.15% CEX)	2.28^A^	99.52

^A–D^Means that do not share the same letter differ (*p* < 0.05). ^1^Total log reduction is obtained by subtracting the initial inoculum from the final log CFU counts that had been obtained on day 28, and the % Log reduction was calculated using the formula, P = (1-(10^-L^)*100), whereby P is percentage log reduction, and L is the Log CFU reduction reported in the table. No statistical analyses were performed on the % Log reduction reported in the table above. WPF, Whey fermentate; CEX, Citrus extract.

### Determination of D-values (decimal reduction times)

There was a difference (*p* < 0.05) in D-values between NC and PC (Table 3). The negative control had a higher D-value (*p* < 0.05) compared to the other treatments except for WPF at 0.25% which was similar to the NC (*p* > 0.05). The D-value of the PC was not different (*p* > 0.05) from 0.25% WPF alone or in combination with CEX at either 0.015% or 0.15%. Additionally, 0.5% WPF alone or in combination with CEX at 0.015% resulted in D-values that were not different (*p* > 0.05) from the PC. However, combination of 0.5% WPF with 0.15% CEX resulted in significantly lower (*p* < 0.05) D-value from the PC but was not different from 1.0% WPF. Overall, increasing the CEX levels from 0.015 to 0.15% in the treatments that contained WPF at 0.25 and 0.5% inclusion levels reduced the D-values compared to when WPF was used alone, although no difference was observed in some instances.

**Table 3:** Decimal reduction times in days (D-values) for *Aspergillus flavus* inoculated in semi-moist pet food containing whey fermentate (WPF) alone or in combination with citrus extract (CEX) 28 days post-inoculation incubated at 25°C.

Treatment	D-Values^1^	SEM
PC (0.1% potassium sorbate)	30.36^CD^	0.296
0.25% WPF	50.98^AB^	0.217
0.5% WPF	25.77^CDE^	0.275
1.0% WPF	12.16^E^	0.383
(0.25% WPF + 0.015%% CEX)	37.09^BC^	0.967
(0.50% WPF + 0.015% CEX)	22.23^CDE^	0.389
(0.25% WPF + 0.15% CEX)	29.49^CD^	0.533
(0.50% WPF+ 0.15% CEX)	15.03^DE^	0.023

^A–D^Means that do not share the same letter differ (*p* < 0.05). ^1^The data used to calculate the D-values were derived from fungal counts of survivors of *A. flavus* inoculated into semi-moist pet food pieces formulated with different inclusion levels of antimycotics for 28 days. Negative Control was emitted from the statistical analysis as it was considered an outlier and it did not contain any WPF or CEX treatments and thus could not be compared with the rest of the treatments. WPF, Whey fermentate; CEX, Citrus extract.

### Shelf life study

**Figure 1:**
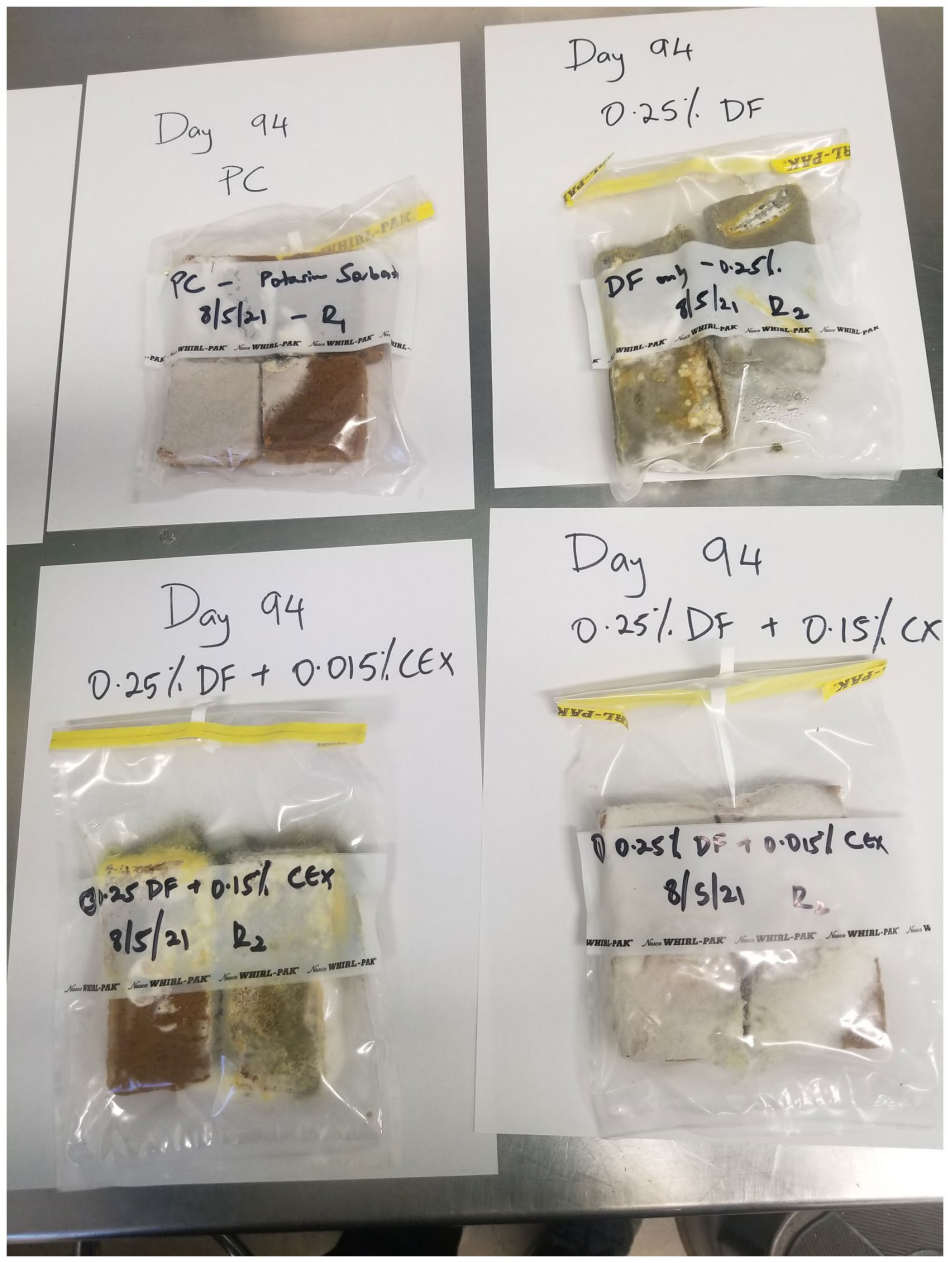
Visible mold growth observed on semi-moist pet food pieces that contained ≤0.5% (w/w) of WPF alone or in combination with CEX at 0.015% or 0.15% after storage at 25°C. DF, Whey fermentate; CEX, Citrus extract; PC, Positive control (treated with potassium sorbate); NC, Negative control (without any antifungal compounds).

**Figure 2:**
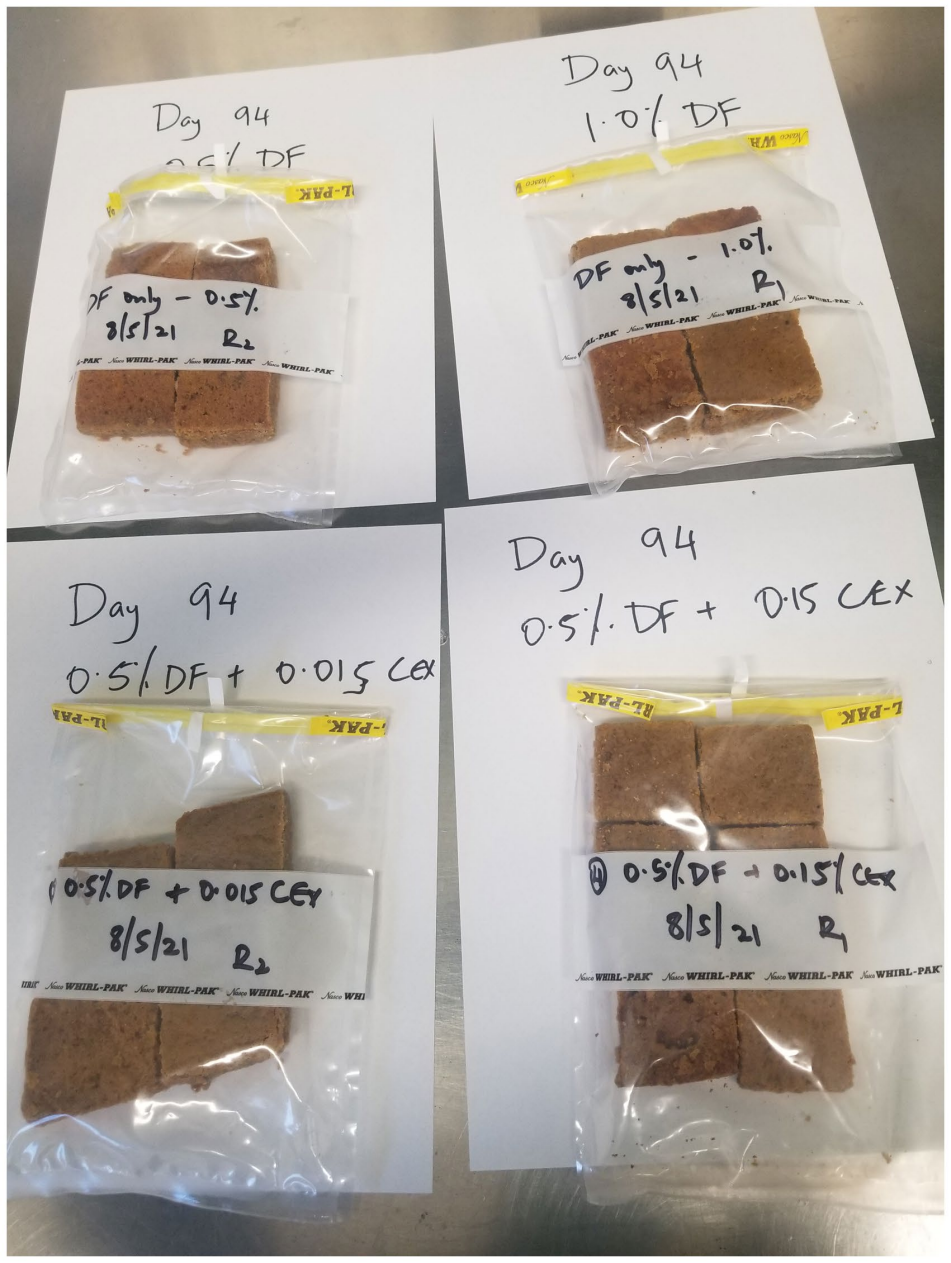
No signs of visible mold growth observed for semi-moist treats containing ≥0.5% (w/w) inclusion levels of WPF alone or in combination with CEX at 0.015% or 0.15% after storage at 25^°^ C. DF, Whey fermentate; CEX, Citrus extract.

The NC samples that contained no antifungal agents were the first to develop mold on the 10th day (Table 4). Treatments containing 0.25% of WPF alone or in combination with CEX at 0.015 and 0.15% exhibited mold growth at days 15, 22, and 38, respectively. The PC only exhibited signs of mold growth after 85 days in storage. The treatments that contained WPF at 0.5% and above irrespective of whether CEX at 0.015% or 0.15% was added to the formula displayed no signs of visible mold growth after 365 days. Figures 1, 2 show images of the molded and unmolded semi-moist pet food taken in this study after 94 days of storage in ambient conditions.

**Table 4:** Total number of days required for visible mold growth to form on semi-moist pet treats containing whey fermentate (WPF) alone or in combination with citrus extract (CEX) to grow visible mold under storage at 25°C.

Treatment level (%)	Days-to-mold
NC	Day 10
PC (0.1% potassium sorbate)	Day 85
0.25% WPF	Day 15
0.5% WPF	No visible mold growth
1.0% WPF	No visible mold growth
(0.25% WPF + 0.015%% CEX)	Day 22
(0.50% WPF + 0.015% CEX)	No visible mold growth
(0.25% WPF+ 0.15% CEX)	Day 38
(0.50% WPF+ 0.15% CEX)	No visible mold growth

PC, Positive control; NC, Negative control; WPF, Whey fermentate; CEX, Citrus extract.

## Discussion

The proliferation of fungi is the most limiting factor in determining the shelf life of semi-moist pet food, as they thrive in conditions of relatively high moisture and water activity. The inclusion of WPF in semi-moist pet food was able to inhibit the proliferation of *Aspergillus flavus*, a challenging toxigenic mold that produces carcinogenic mycotoxins such as aflatoxin B1, as the preliminary study reported by Kiprotich et al. (2021) showed.

The process of fermenting whey using LAB produces bioactive compounds such as peptides with antifungal properties. For instance, Luz et al. (2020) reported that phenolic compounds which possess antifungal properties had been isolated in a cell-free supernatant that had been manufactured by fermenting whey using a LAB strain, *Lactobacillus plantarum*. The phenolic compounds produced by the fermentation of whey using *L. plantarum* were hydroxybenzoic acid, phenyllactic acid, and lactic acid that impeded the growth of Mucor spp., Penicillium digitatum, and *Bacillus subtilis* (Barman et al., 2017). Dal Bello et al. (2007) demonstrated that fermentation of whey using LAB produced cyclic dipeptides (L-Leu-L-Pro and L-Phe-L-Pro) in culture media that were used to extend the shelf life of sourdough bread.

In the current study, spray dried WPF was added to the semi-moist pet food at different inclusion levels and mixed with water before thermal processing. The hypothesis was that the hydration effect of water added to the batter activated the antifungal compounds present in WPF which impeded the proliferation of *A. flavus*. However, the maximum amount of WPF that could be added to the formula without compromising the organoleptic properties of the semi-moist pet food was 1.0% (w/w). This was evident as inclusion levels above 1.0% exhibited superior antifungal effects but resulted in a poorly formed product. Increasing the inclusion levels of WPF from 0.25 to 1.0% resulted in increased log reduction but resulted in increased costs of production upon product formulation. Currently, there is limited data that show safety or toxicity effects of WPF or CEX on animal health, however, increasing these compounds in semi-moist pet food had adverse effects on the sensory and palatability of the product.

Citrus extracts obtained from citrus fruit peels or leaves have demonstrated strong antifungal properties against fungal strains such as *P. digitatum* and *P. italicum* (Trabelsi et al., 2016). Analysis of citrus extract obtained from peels showed that these extracts contained a wide range of flavonoids in high quantities, coumarins and volatile compounds such as phytoalexins that inhibit proliferation of pathogenic fungi (Chen et al., 2019). However, preliminary studies by Kiprotich et al. (2021), reported that CEX exhibited low antifungal activity when used as a mold inhibitor in semi-moist pet treats. Moreover, the product manufacturer reported that the maximum daily allowable daily intake of CEX is 0.15%, which failed to exhibit any antimycotic effect in semi-moist pet food. Preliminary studies also demonstrated evidence of potentiation as CEX alone exhibited limited to no antimycotic activity when used alone as a mold inhibitor but resulted in increased log reductions when it was combined with WPF, compared to when WPF was applied alone. Nonetheless, additional studies are warranted to investigate the type and quantity of antimicrobial compounds present in the WPF. The composition of CEX that was used in this study also needs to be investigated so that the synergistic antifungal compounds present in this extract responsible for potentiating WPF are understood such that the mechanisms of mycotic inhibition of these two compounds can be investigated.

D-values refer to the time (days) required for an antifungal (potassium sorbate, WPF alone, or WPF in combination with CEX) to reduce the initial population of *A. flavus* by 90% or 1.0 log CFU/piece. The D-values obtained in this study provide insights into the fungicidal and/or fungistatic nature of the treatments, as they estimate the amount of time required to achieve a 90% kill of the *A. flavus* inoculated in the semi-moist pet food.

The treatments that contained WPF at 1.0% alone or WPF at 0.5% combined with CEX at 0.15% had the most fungicidal effect, evidenced by the fewer number of days required to achieve a 1.0 log kill of the pathogenic mold. Treatments that contained 0.5% WPF alone or in combination with CEX at 0.015% were more fungistatic compared to the treatments that contained WPF at 0.25% alone or in combination with CEX at 0.015% or 0.15%. The WPF at 0.25% alone or in combination with CEX at 0.015% or 0.15% were completely overwhelmed by opportunistic spoilage mold by day 28, even though the log CFU counts of the *A. flavus* had reduced from the initial concentration. The PC (0.1% potassium sorbate) had a more fungistatic effect, even though the D-values observed from this treatment were high and comparable to treatments at 0.25% WPF alone or in combination with CEX at 0.015 and 0.15%. The PC was fungistatic because it did not show any signs of visible mold spoilage until day 85, compared to the latter treatments. The D-values obtained from this study followed a similar trend to that of the log reductions. For instance, the NC had the highest D-value compared to the PC and the rest of the treatments. The treatments containing 0.5% WPF when combined with 0.15 and 0.015% of CEX yielded numerically lower (*p* > 0.05) D-values as compared to other combination treatments as well as 0.25 and 0.5% WPF individually. This finding could be indicative of potentiation between the two treatments in inhibiting mold in semi-moist pet food. However, the D-values for NC, 0.25% WPF alone or in combination with CEX at 0.015% or 0.15%, might be misleading as these semi-moist pet foods that had been treated with these treatments were overwhelmed by opportunistic spoilage mold that may have outcompeted *A. flavus*. Thus, these values are not evidence of mold inhibition by these treatments. However, additional studies are warranted to study the antifungal (fungicidal and fungistatic) effects of WPF and CEX alone or in combination using microdilution to evaluate fungal conidial germination or the disc diffusion assay, which were not possible in this study because of the powdery nature of WPF that formed colloidal suspensions and the partial solubility of the CEX in water, which complicated these assays.

The shelf life experiments investigated the mold inhibitory effects of the treatments. Semi-moist pet foods are high in moisture and have a water activity that favors the growth of toxigenic and spoilage fungi. Fermentation of whey proteins produce phenolic compounds that inhibit the growth of fungi, for instance, Izzo et al. (2020a) replaced water with whey fermented using *L. plantarum* during baking and that significantly increased the shelf life of pita bread. Luz et al. (2018) evaluated phenolic compounds that were present in freeze-dried whey fermented with *L. plantarum* strains 220 and 221 for 72 h. Their analysis revealed a significant number of benzoates (hydroxybenzoic acid, benzoic acid, 1,2-dihydroxibenzene) which possess significant antifungal activity as evidenced by their industrial application in foods for mold inhibition. Luz et al. (2018) also reported a significant number of polyphenols with demonstrated antifungal properties such as sinapic acid, DL-3-phenillactic acid, caffeic acid, p-coumaric acid, salicilic acid, and hydrocinnamic acid. Collectively, these benzoates and polyphenols can be hypothesized as inflicting stressors on fungi, creating multiple hurdles thus eventually inhibiting their growth or inactivating them. In this present study, it took the NC treatment only 10 days for signs of visible mold growth to be observed. The WFP treatments at 0.25% alone or in combination with CEX at 0.015 and 0.15% exhibited visible signs of mold growth in 15, 22 and 38 days, respectively. The PC (0.1% potassium sorbate) treatment seemed to be moderately effective at preventing mold growth. This is because visible signs of mold on the PC samples were observed on day 85. The rest of the treatments with WPF at inclusion levels of >0.5% (w/w) alone or in combination with CEX did not show any signs of visible mold growth even after 365 days of storage in ambient conditions. Our findings from the shelf life study suggest that WPF has significantly superior fungistatic effects compared to potassium sorbate, albeit its combination with CEX potentiates this effect, ultimately increasing the shelf life of semi-moist pet food. Thus, the rationale of pairing WPF with CEX is to create a potentiation effect which in turn reduces the dose of WPF required to control mold which lowers production costs in the pet food industry. Additional studies are warranted to identify the type and concentration of the phenolic and benzoic acids present in WPF to better explain the mechanism of fungal inhibition.

## Conclusion

The contamination and proliferation of spoilage and toxigenic fungi in semi-moist pet foods is a perennial challenge for manufacturers, exacerbated by the ever-increasing demand for products that are considered “natural” and have “clean labels” as healthier alternatives for feeding companion animals. The utilization of whey fermented with lactic acid bacteria strains such as *L. plantarum* can be used to produce value-added and “natural” antifungal compounds that can be used as alternative substitutes to synthetic preservatives such as potassium sorbate to control mold in semi-moist pet foods and baked goods intended for human consumption. However, because larger doses of fermented whey are required to achieve the desired level of microbial safety in semi-moist pet foods, additional research into polyphenols with potentiating or synergistic antimycotic effects is warranted to boost the applicability of WPF. This current study creates a scientific framework for the application of fermented whey protein into semi-moist pet food as this ingredient gains in popularity among pet food brands that sell “natural” and clean label products. In conclusion, WPF at 0.5–1.0% (w/w) inclusion levels can be used alone or in combination with CEX as natural antifungal treatments to control spoilage and toxigenic fungi in semi-moist pet foods for dogs.

## Data availability statement

The raw data supporting the conclusions of this article will be made available by the authors, without undue reservation.

## Author contributions

GA, JD, and CR conceived the idea, designed the study, helped with editing, and interpretation of results. SK performed all the experiments, collected data, data analysis, and wrote the primary manuscript. All authors contributed to the article and approved the submitted version.
